# Supplementary material for: Kalkitoxin: A Potent Suppressor of Distant Breast Cancer Metastasis
Source: Int J Mol Sci. 2023 Jan 7;24(2):1207. doi: 10.3390/ijms24021207 (PMC9863388; doi:10.3390/ijms24021207)
Supplement: Supplementary file 1 [file ijms-24-01207-s001.zip › ijms-2077414-supplementary.pdf]

A

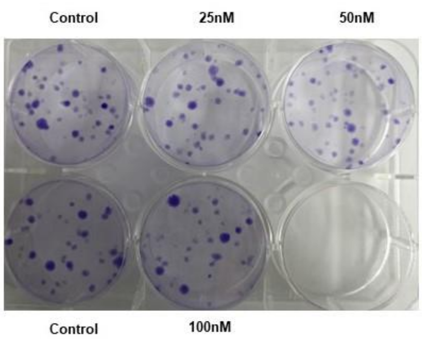

B

Concentration of KT	Colony
0 nM	42
25 nM	40
50 nM	41
100 nM	37

C

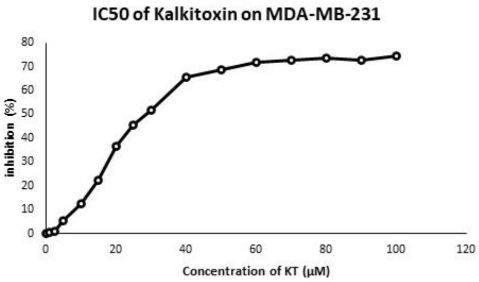

Supp Fig. S1. Colony formation assay and IC_{50} of kalkitoxin. (A) Cells were cultured with DMEM high glucose with or without KT of 25, 50, and 100 nM separately for 5 days. (B) Number of colonies formation with or without treatment of KT. (C) Cells were treated with different concentrations of KT for 24 hours and IC_{50} was calculated.
